# FGF9 can induce endochondral ossification in cranial mesenchyme

**DOI:** 10.1186/1471-213X-6-7

**Published:** 2006-02-20

**Authors:** Venkatesh Govindarajan, Paul A Overbeek

**Affiliations:** 1 Cancer Center, Creighton University, Omaha, NE 68178, USA; 2 Department of Molecular and Cellular Biology, Baylor College of Medicine, Houston, TX 77030, USA

## Abstract

**Background:**

The flat bones of the skull (i.e., the frontal and parietal bones) normally form through intramembranous ossification. At these sites cranial mesenchymal cells directly differentiate into osteoblasts without the formation of a cartilage intermediate. This type of ossification is distinct from endochondral ossification, a process that involves initial formation of cartilage and later replacement by bone.

**Results:**

We have analyzed a line of transgenic mice that expresses FGF9, a member of the fibroblast growth factor family (FGF), in cranial mesenchymal cells. The parietal bones in these mice show a switch from intramembranous to endochondral ossification. Cranial cartilage precursors are induced to proliferate, then hypertrophy and are later replaced by bone. These changes are accompanied by upregulation of *Sox9*, *Ihh*, *Col2a1*, *Col10a1 *and downregulation of *CbfaI *and *Osteocalcin*. Fate mapping studies show that the cranial mesenchymal cells in the parietal region that show a switch in cell fate are likely to be derived from the mesoderm.

**Conclusion:**

These results demonstrate that FGF9 expression is sufficient to convert the differentiation program of (at least a subset of) mesoderm-derived cranial mesenchyme cells from intramembranous to endochondral ossification.

## Background

Bone development can occur in two distinct ways: 1) through endochondral ossification where the mesenchymal cells differentiate into chondrocytes and lay down a cartilaginous template that is later replaced by bone; or 2) through intramembranous ossification where mesenchymal cells directly differentiate into osteoblasts without the formation of a cartilage intermediate. During endochondral ossification, the transcription factors Sox9, Sox5 and/or Sox6 are expressed and are involved in the induction of chondrocytes [1]. Chondrocytes in the growth plate are subsequently induced to exit the cell cycle and commit to terminal differentiation. The prehypertrophic chondrocytes mature into hypertrophic chondrocytes, which lay down a matrix rich in Collagen X, and secrete VEGF [2]. VEGF promotes the invasion of blood vessels from the perichondrium, bringing in both the bone forming osteoblasts and the bone resorbing osteoclasts. The hypertrophic chondrocytes then undergo apoptosis, and are replaced by trabecular bone and bone marrow. In contrast, the flat bones of the skull, the frontal and parietal bones, form by intramembranous ossification. Cranial mesenchymal cells directly differentiate into osteoblasts that initiate mineralization and secrete an extracellular matrix rich in Collagen I [3]. Growth of these calvarial bones occurs through proliferation and differentiation of osteoblasts at the margins or sutures. The molecular pathways that dictate these alternative ossification programs are not yet well defined. In particular, it is not known whether intramembranous ossification is prespecified by the ontogeny of the cranial mesenchyme or is a response to local environmental signals.

Fibroblast growth factors (FGFs) appear to be important for both types of ossification [4]. FGFs comprise a large family of proteins that includes at least 22 known members [5]. FGFs bind and signal through low and high affinity FGF receptors [5]. The four known high affinity receptors (FGFR1–4) are structurally similar transmembrane receptor tyrosine kinases. During intramembranous ossification of the flat bones, FGFR1–3 are expressed by the differentiating osteoblasts at the osteogenic fronts and also by the adjacent cartilage [6,7]. FGFR1 is expressed in cells close to and within the osteoid; FGFR2 is expressed in the proliferating osteogenic stem cells; FGFR3 expression is seen in the thin layer of cartilage underlying the lower part of the coronal suture [6]. As FGFR3 null mice do not show defects in calvarial development, it has been hypothesized that intramembranous bone formation is controlled primarily by FGFR1 and FGFR2 [8]. Mutations in FGFR1, FGFR2 and FGFR3 in humans that affect skeletal growth are consistent with this hypothesis [9]. Craniosynostosis is mainly associated with mutations in FGFR1 and FGFR2 [9]. Mutations that affect the growth of long bones resulting in syndromes such as Achondroplasia (Ach) and Thanatophoric dysplasia (TD) are mainly localized to FGFR3. These autosomal dominant disorders are believed to reflect either an enhancement of receptor activity or a neomorphic gain-of-function effect [9-11]. During long bone development, FGF receptors are expressed in the epiphyseal growth plates: FGFR3 is expressed in the proliferating chondrocytes; FGFR1 is expressed in the hypertrophic chondrocytes; FGFR1 and FGFR2 are expressed in the perichondrium [4,12]. FGFR2 is expressed in early mesenchymal condensates and in the periosteal collar around the cartilage models [8]. Targeted deletion of FGFR2IIIc suggests that FGFR2IIIc is a positive regulator of ossification in both the osteoblasts and chondrocytic lineages [13]. Targeted deletion of FGFR3 results in mice that show overgrowth of the long bones and abnormal proliferation of chondrocytes suggesting that FGFR3 stimulation inhibits chondrocyte proliferation [14]. These studies describe roles for FGF receptor-mediated signaling during differentiation/maturation of chondrocytes.

The roles of FGF ligands in skeletal development are unclear. During calvarial development, *Fgf8 *is expressed in the osteoblasts; *Fgf2 *and *Fgf4 *are expressed in the sutural mesenchyme; *Fgf18 *is initially expressed in the cranial mesenchymal cells and later, in the differentiating osteoblasts [8]. *Fgf9 *is expressed in the sutural mesenchyme and is upregulated in the endocranial portions of the mesenchyme and is downregulated during postnatal development [15][16]. The specific in vivo functions of these different FGFs remain undefined. In vitro, cephalic neural crest cells from quail embryos have been shown to respond to exogenous FGF-2 in a dose dependant manner; lower doses induce proliferation and higher doses induce cartilage differentiation [17]. During long bone development, at the time of initiation of endochondral differentiation, *Fgf9 *is expressed in the condensing mesenchyme [18]. *Fgf2*, *Fgf5*, *Fgf6 *and *Fgf7 *are expressed in loose mesenchyme outside the condensation [19-23]. However, mice lacking these Fgfs show no apparent defects in skeletal development [24-28]. Functional redundancy between these FGFs may, in part, account for the lack of phenotype. Therefore, roles for these Fgfs in the early stages of chondrogenesis have not yet been defined.

In this study, we have analyzed transgenic mice that express FGF9 in their cranial mesenchymal cells. These mice show abnormal head development and are born with a pronounced bulge in their skulls. Skeletal preparations of these mice revealed dramatic changes in parietal bone formation. In the region where the parietal bones normally form, cranial mesenchymal cells are induced to differentiate into chondrocytes that proliferate, hypertrophy and subsequently differentiate as bone. Correlative changes in expression of marker genes *Sox9*, *Col2a1*, *Col10a1*, *Ihh*, *CbfaI *and *Osteocalcin *occur in conjunction with the altered differentiation program. Thus the parietal bones in these mice form by endochondral ossification rather than by the usual intramembranous ossification route. Fate mapping studies indicate that the ectopic cartilagenous precursors in these mice are derived from the mesoderm. Based on these results we suggest that cranial mesenchymal cells are competent to initiate endochondral ossification, and can be switched to this alternative developmental program by ectopic expression, or overexpression, of FGF9.

## Results

### Changes in cranial morphology in the FGF9 transgenic mice

**Figure 1:**
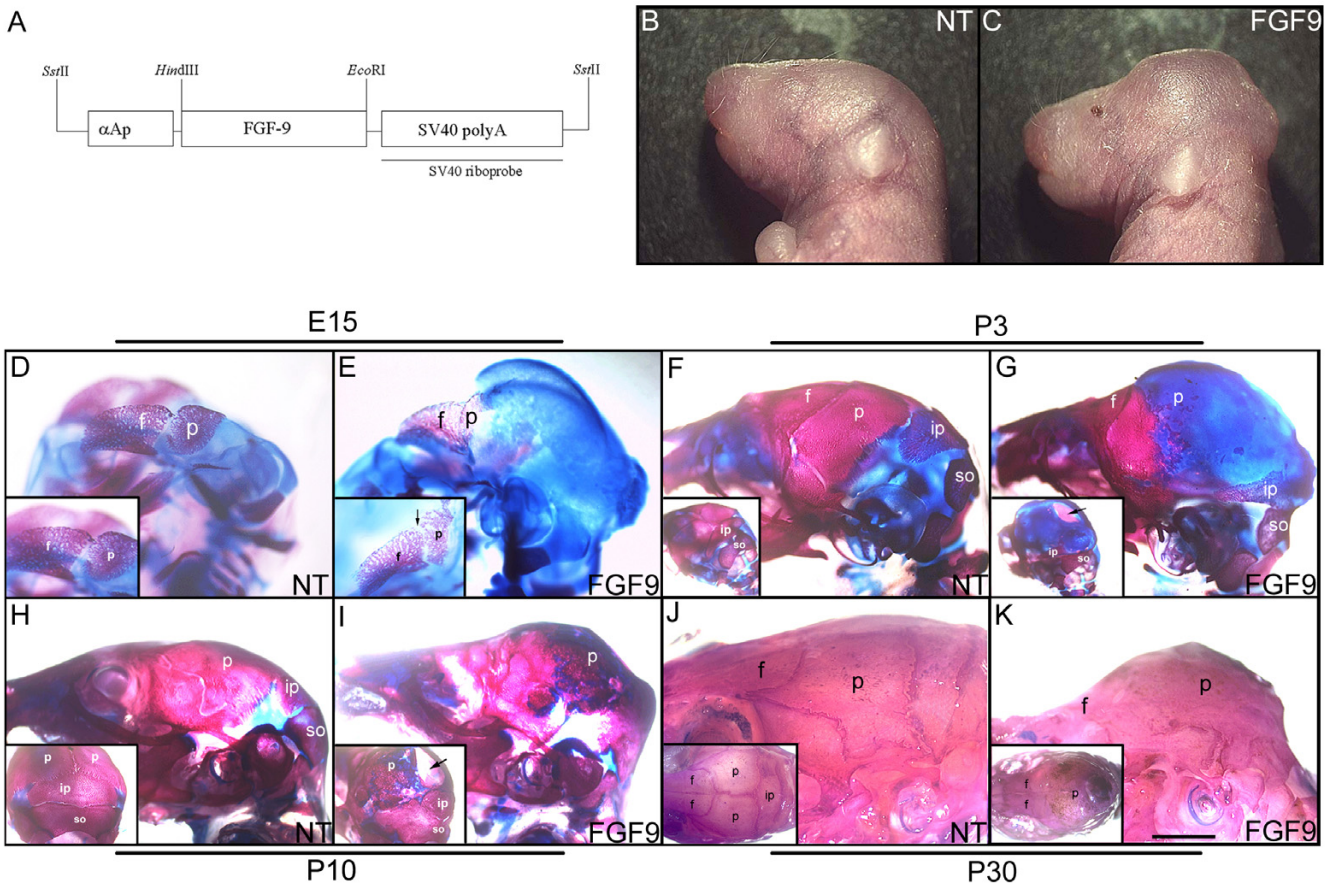
Abnormal head development in the OVE1070 FGF9 transgenic mice. Panel A shows a schematic representation of the FGF9 transgene. The coding region of mouse *Fgf9 *cDNA was inserted between the αA-crystallin promoter (αAp) and an intron and polyadenylation sequence derived from SV40 virus [47]. The microinjection fragment was generated by *Sst*II digestion. The SV40 sequences were used to make a riboprobe for detection of expression of the transgene. (B and C) Three day old nontransgenic (NT) and FGF9 transgenic mice are shown. The heads of the transgenic mice are 'dome' shaped (C). The OVE1070 transgenic mice often show unfused eyelids at birth (C). (D-K) Skeletal preparations of the FGF9 transgenic mice reveal an expansion in the cartilage territory in the head region. Alcian blue and Alizarin red stained E15 (D and E), P3 (F and G), P10 (H and I) and P30 (J and K) heads are shown. Cartilage is stained blue and bone red. The insets in panels D and E are higher magnifications of the frontal and parietal bones in the nontransgenic and transgenic mice respectively. The insets in panels F-I are rear views. The insets in panels J and K are top views. An expansion in the cartilage territory is seen in the FGF9 transgenic mice. Parietal bone (p) is affected in the transgenic skulls but the coronal suture is still present (E inset, arrow). Frontal (f) and occipital bone formation appear to be unaffected (E and G insets). A hole forms in the skull of the FGF9 transgenic mice that is visible by P3 (G inset, arrow) and is seen to persist at P10 (I inset, arrow). The red stain visible at the arrow in the inset in panel G is bone at the base of the head, which is visible since the skull and the brain are transparent after staining and clearing. The cartilage territory is replaced by bone by P30 (K and inset). Scale bar: 1 mm in D and E; 1.6 mm in F and G; 2.5 mm in H-K.

Transgenic mice were generated by microinjection of a construct with the αA-crystallin promoter linked to a mouse *Fgf9 *cDNA (Fig. 1A) [29]. Eleven founders carrying the transgene were identified. Five of them had cataracts (data not shown). In the transgenic line OVE1070, in addition to lens defects, there were defects in the development of the skull (Fig. 1B, C). Mice in this family that are heterozygous for the transgene are born with 'dome-shaped' heads (Fig. 1C). This phenotype is even more pronounced in homozygous transgenic mice (data not shown).

### Calvarial development in FGF9 transgenic mice

The skeletal defects were examined by Alcian blue and Alizarin red staining of E15, P3, P10 and P30 mice (Fig. 1D–K). The skeletal preparations show a dramatic expansion of cartilage in the head region by E15 (compare Fig. 1E to 1D). Parietal bone development was particularly affected (Fig. 1E and inset). At P3, the phenotypic differences between the nontransgenic and FGF9 transgenic mice were still pronounced. At this age, the calvarial skull in nontransgenic mice is mostly ossified except at the sutures (Fig. 1F). In contrast, there is an expanded cartilage territory (blue) in the FGF9 transgenic mice (Fig. 1G). The transgenic mice also show a central hole in the skull where there is neither cartilage nor bone (Fig. 1G, inset, arrow). Development of the occipital bones appeared normal (Fig. 1G and inset). At P10, the Alcian blue stained tissues in the transgenic cranium were interspersed with Alizarin red stained regions suggesting that the cartilage territory was being replaced by bony structures (Fig. 1I). By 1 month of age, the cartilage territory was completely replaced by bone (Fig. 1K and inset).

**Figure 2:**
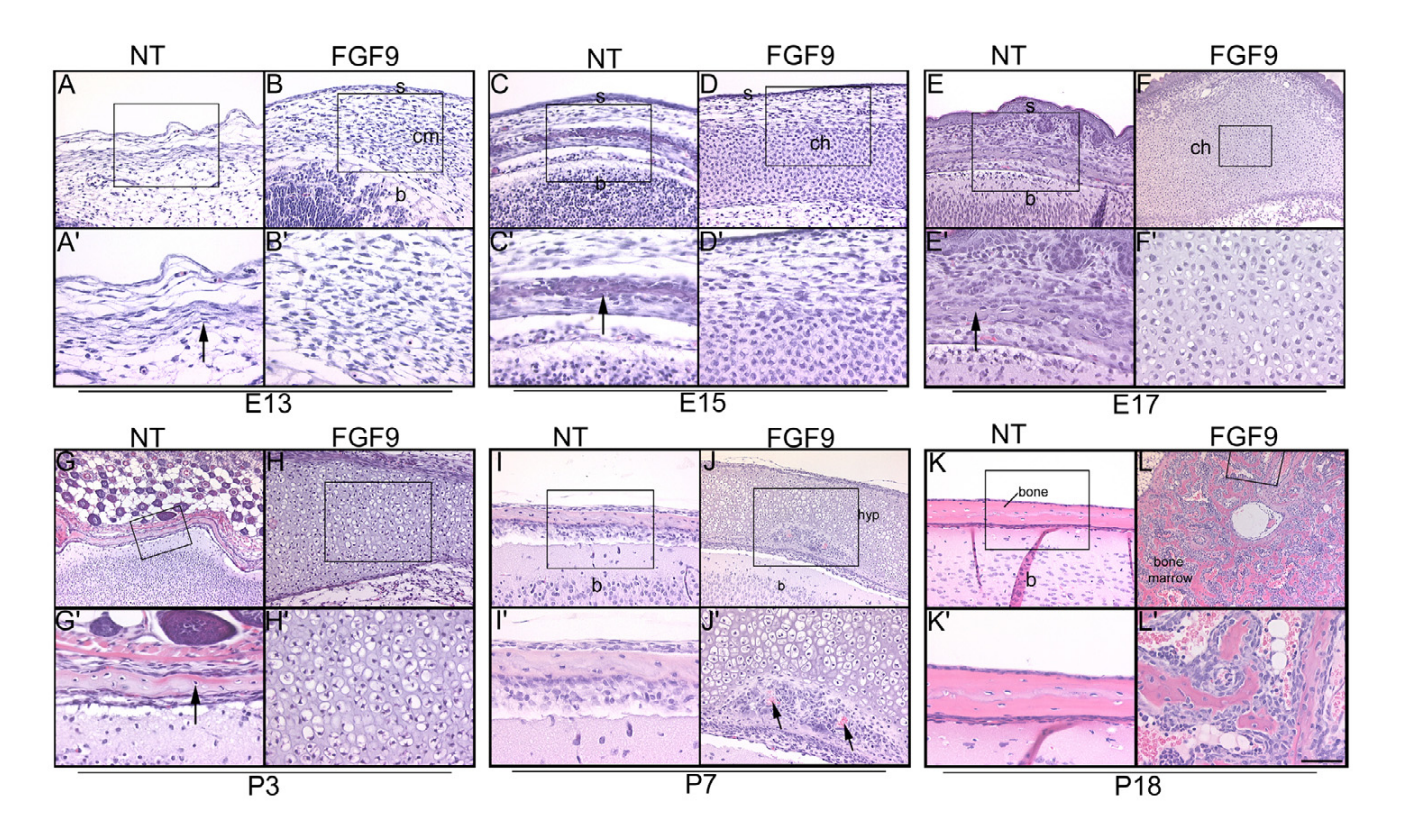
Cranial mesenchyme in the FGF9 transgenic mice differentiates into chondrocytes. Heads of nontransgenic (A, A', C, C', E, E', G, G', I, I', K and K') and FGF9 transgenic mice (B, B', D, D', F, F', H, H', J, J', L and L') were sectioned and stained with hematoxylin and eosin. Panels A'-L' are higher magnifications of the boxed regions in panels A-L respectively. In nontransgenic mice, the mesenchymal cells of the skull form a skeletogenic membrane (arrows in A', C', E' and G') within which the bones form. In the FGF9 transgenic mice the cranial mesenchymal cells (cm) differentiate into chondrocytes (ch) that initially form a structure resembling the hyaline cartilage (D, D', F and F') and later hypertrophy (hyp) (H, H', J and J'). Perichondrial cells, including blood cells (J', arrows) invade and replace the hypertophic chondrocytes forming bone and bone marrow (L and L'). Other abbreviations: b, brain; s, skin. The folding seen in the section in panel G is an artifact of the histology procedure. Note that the skin was removed from P7 and P18 mice to facilitate fixation and histological processing. Scale bar: 50 μm in A'-L'; 100 μm in A, B, C, D, E, H, I and K; 200 μm in F, G, J and L.

The alterations in skeletal development in the FGF9 transgenic mice were examined at the histological level by analyses of hematoxylin and eosin stained sections of the cranium. No distinctive differences in morphology were seen at E11 (data not shown). By E13, more cranial mesenchymal cells were seen in the skull region of the FGF9 transgenic mice (Fig. 2B, B') and these cells were organized differently from nontransgenic mice (compare Fig. 2A, B). By E15, the cranial mesenchymal cells in the nontransgenic mice had initiated intramembranous ossification (Fig. 2C, C', arrow). In contrast, the mesenchymal cells in the FGF9 transgenic skulls differentiated into cells resembling chondrocytes (Fig. 2D, D'). Within the next few days these cells increased in size and became hypertrophic (Fig. 2F, F', H, H'). By P7, perichondrial cells (including the blood vessels) had begun to invade the cartilage territory (Fig. 2J, J'). By P18, the cartilage territory was transformed into trabecular bone and bone marrow (Fig. 2L, L'). These results considered together suggest that the cranial mesenchymal cells in the parietal region in these mice recapitulate the sequence of events that occurs during endochondral ossification.

### Ectopic expression of the FGF9 transgene

**Figure 3:**
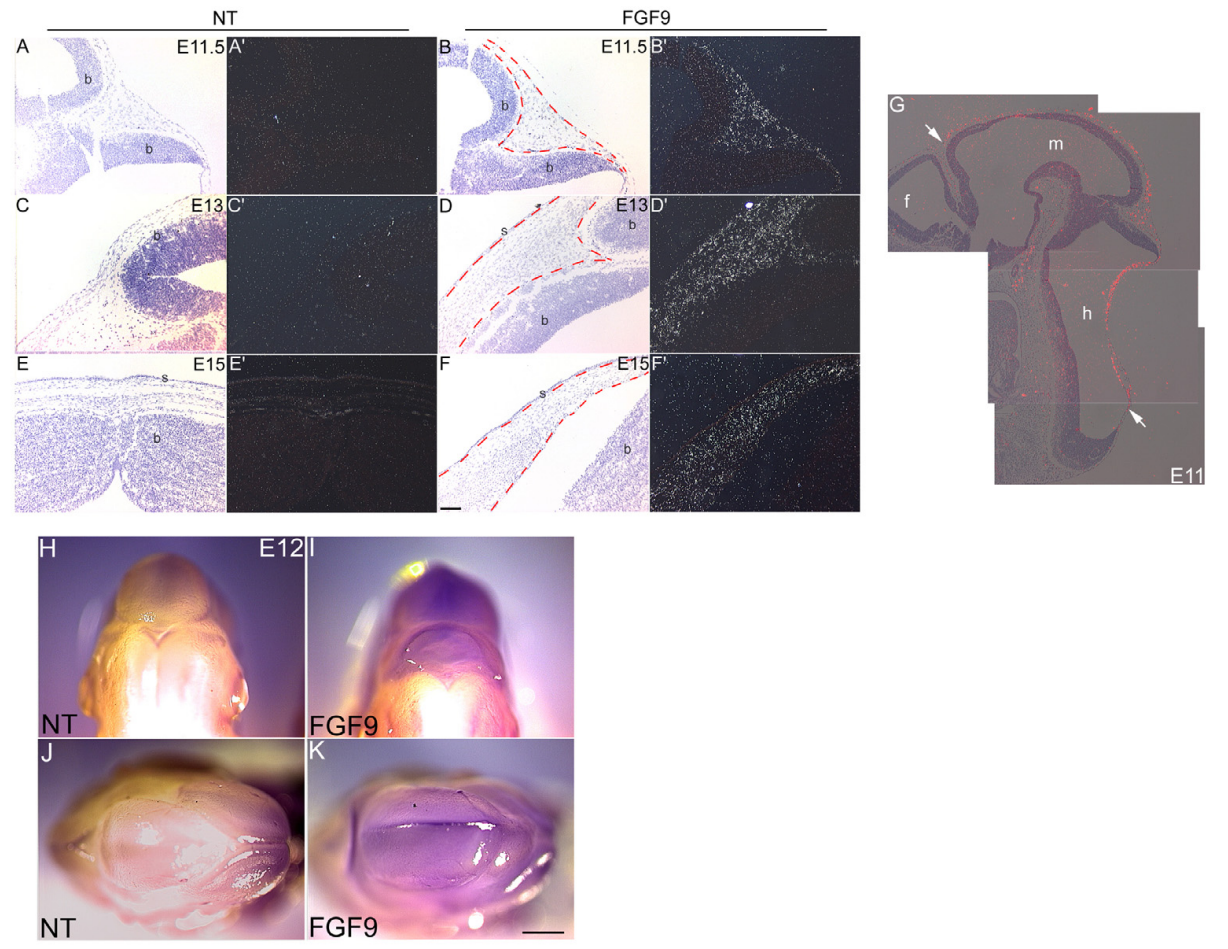
Expression pattern of the transgene. In situ hybridizations were done on sections of heads from nontransgenic (NT) and FGF9 transgenic mice using a [^35^S]-labeled SV40 riboprobe. Panels A-F show bright-field images and panels A'-F' show the corresponding dark field images. Transgene expression can be seen in the cranial mesenchymal cells at E11 (B'), E13 (D') and E15 (F'). At later stages, expression of the transgene in the cranium was not detected (data not shown). Transgene expression was restricted to the mesenchymal cells overlying the mid (m) to hind brain regions (h) but not the forebrain (f) (G, arrows). Scale bar: 100 μm in A-F'. Whole mount in situ hybridizations on E12.5 heads using a digoxygenin-labelled SV40 riboprobe show expression of the transgene (purple color) in the cranial regions (H-K). Panels H and I are rear views and panels J and K are top views. Scale bar: 1 mm in H, I and K.

To test if the alterations seen in the skulls of the OVE1070 mice were due to ectopic expression of the transgene, in situ hybridizations were performed using an S^35^-labelled riboprobe that recognizes the SV40 portion of the transgene (Fig. 1A). Transgene expression was seen in the lens [29], the dorsal portion of the retinal pigmented epithelium [30] and also in the cranial mesenchymal cells beginning at E11 (Fig. 3B', D', F'). Expression levels peak by E13 and start to decrease by E15 (Fig. 3D', F'). Transgene expression is extinguished by E17 (data not shown). Section and whole mount in situ hybridizations show that the extraocular transgene expression is restricted to the mesenchymal cells overlying the future mid and hind brain regions (Fig. 3G, arrows; 3H-M). These results argue that the defects in skeletal development in the OVE1070 line are attributable to ectopic and transient expression of the FGF9 transgene.

### Expression of chondrocyte specific markers

**Figure 4:**
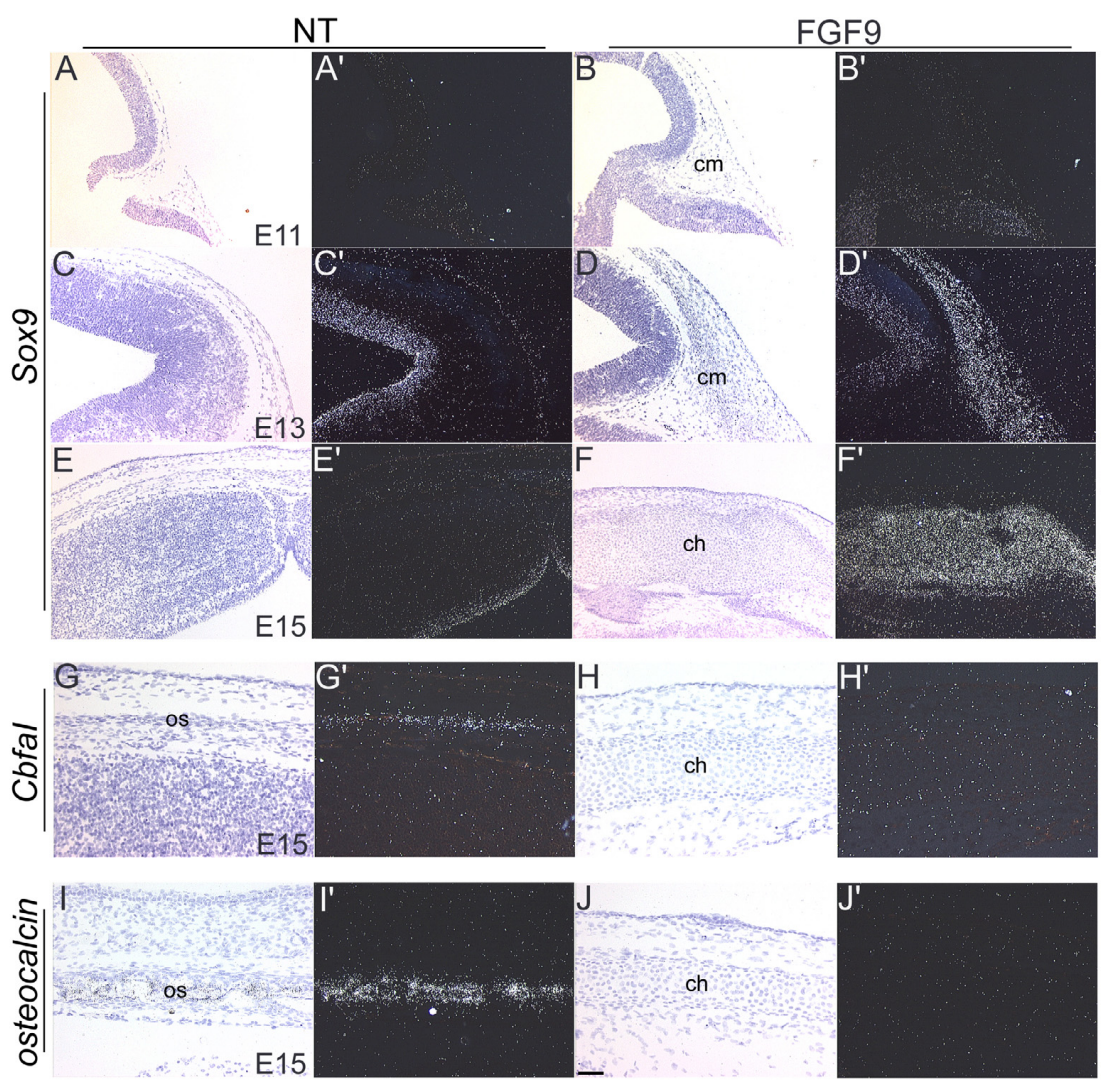
Expression patterns of *Sox9*, *CbfaI *and *Osteocalcin*. In situ hybridizations were done on sections of heads from nontransgenic (NT) and FGF9 transgenic mice using [^35^S]-labeled riboprobes for *Sox9 *(A-F'), *CbfaI *(G-H') and *osteocalcin *(I-J'). Panels A-J are bright field images and panels A'-J' are the corresponding dark-field images. Expression of *Sox9 *is induced in the cranial mesenchymal cells of the FGF9 transgenic mice by E13 (D'). *Sox9 *expression is detected in the ventricular regions of the brain at E13 in both the transgenic and nontransgenic mice (C', D'). *CbfaI *and *osteocalcin *are expressed in the differentiating calvarial osteoblasts (os) of nontransgenic mice at E15 (G', I') but not in the skulls of the FGF9 transgenic mice (H', J'). Scale bar: 50 μm in G-J'; 100 μm in A-F'.

**Figure 5:**
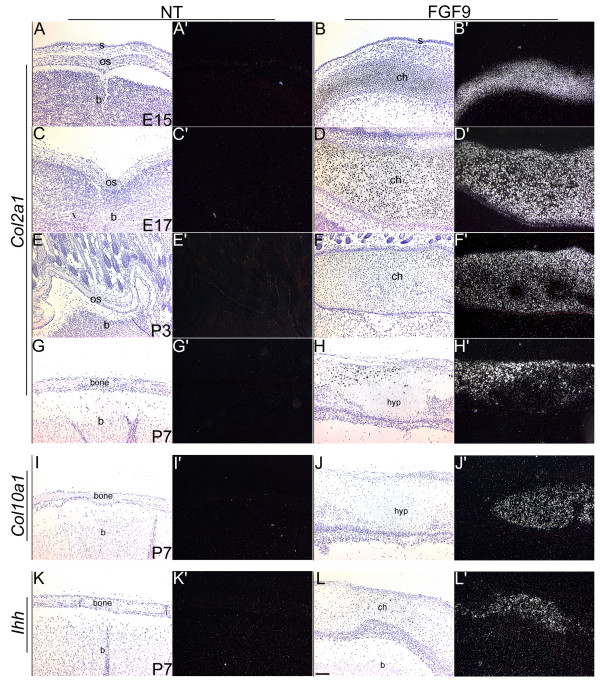
Expression patterns of *Col2a1*, *Col10a1 *and *Ihh*. In situ hybridizations were done on sections of heads from nontransgenic (NT) and FGF9 transgenic mice using [^35^S]-labeled riboprobes for *Col2a *(A-H'), *Col10a1 *(I-J') and *Ihh *(K-L'). Panels A-L are bright field images and panels A'-L' are the corresponding dark-field images. Expression of *Col2a1 *is enhanced in the cranial mesenchymal cells of the FGF9 transgenic mice by E15 (B') and is decreased in some cells by P7 (H'). Hybridizations performed on adjacent sections show expression of *Col10a1 *in these hypertropic (hyp) chondrocytes (J'). Ihh expression was detected in the differentiated chondrocytes by P7 (L'). Abbreviations: b, brain; ch, chondrocytes; hyp, hypertropic chondrocytes; os, osteoblasts; s, skin. Scale bar: 100 μm in A-L'.

The different stages of endochondral ossification are characterized by expression of different marker genes. Expression of *Sox9*, a member of the Sox family of transcription factors, has been shown to be essential for chondrocyte condensation [31]. In particular, *Sox9 *is required for the expression of cartilage-specific extracellular matrix components such as Collagen II (*Col2a1*) [32]. In the long bone growth plates, *Col2a1 *is expressed in the resting and proliferating chondrocytes, *Ihh *in prehypertropic chondrocytes and *Col10a1 *in hypertropic chondrocytes [3]. Expression of the runt domain transcription factor *CbfaI *has been shown to be essential for differentiation of the cells in the osteoblastic lineage [33]. Osteocalcin, one of the downstream targets of *CbfaI*, is expressed exclusively in the osteoblasts [34]. Expression of these six marker genes was examined by in situ hybridization using S^35^-labelled riboprobes (Figs. 4 and 5). No appreciable differences in expression of *Sox9 *between nontransgenic and FGF9 transgenic mice were seen at E11 (Fig. 4A, A', B, B'). However, significant induction of *Sox9 *expression was seen in the cranial mesenchymal cells of the transgenic mice by E13 (Fig. 4C, C', D and 4D'). *Sox9 *expression was seen to persist in the developing chondrocytes (Fig. 4F, F'). *CbfaI *and *Osteocalcin *are expressed during the normal program of intramembranous ossification in nontransgenic mice, but their expression was not seen in the corresponding regions of the embryonic transgenic heads (Fig. 4G–J').

*Col2a1 *expression was seen in the developing chondrocytes in the transgenic cranium but not the wildtype, at E15, E17 and P3 (Fig. 5A–F'). By P3 expression levels were found to be reduced in transgenic hypertropic chondrocytes (Fig. 5E–H'). In situ hybridizations at P7 showed that *Col10a1 *and *Ihh *are expressed in the hypertropic chondrocytes (Fig. 5I–L'). Expression of *Col10a1 *was not seen in the chondrocytes at P3 or before (data not shown). These results demonstrate that the transgenic cranial mesenchymal cells undergo endochondral ossification instead of intramembranous ossification.

### Expression of *Fgf9*, *Fgfr2 *and *Fgfr3*

**Figure 6:**
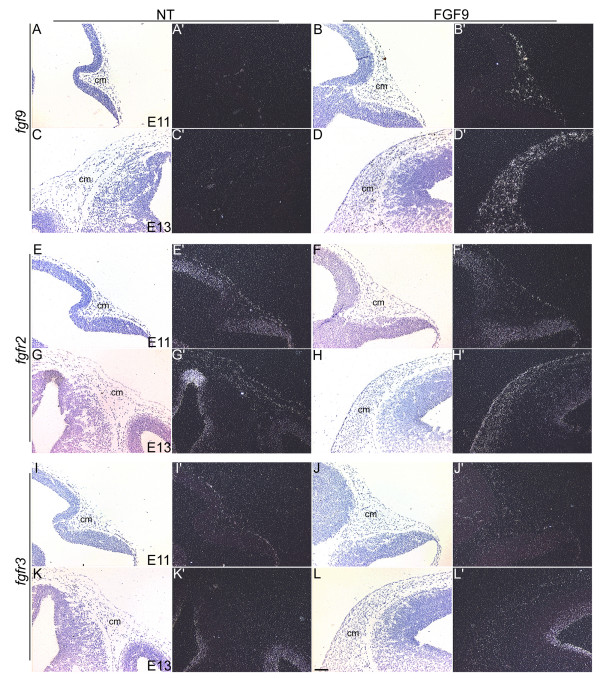
Expression patterns of *Fgf9*, *Fgfr2 *and *Fgfr3*. In situ hybridizations were done on sections of heads from nontransgenic (NT) and FGF9 transgenic mice using [^35^S]-labeled riboprobes for *Fgf9 *(A-D'), *Fgfr2 *(E-H') and *Fgfr3 *(I-L'). Panels A-L are bright field images and panels A'-L' are the corresponding dark-field images. Expression of *Fgf9 *is not seen in the cranial mesenchymal (cm) cells of the nontransgenic mice at E11 (A') or E13 (C') in contrast to age-matched sections of FGF9 transgenic heads (B' and D'). Modest *Fgfr2 *expression is detected in the cranial mesenchymal cells at E11 in both the transgenic and nontransgenic mice (E' and F') and persists in the expanded cranial mesenchyme at E13 (H'). *Fgfr3 *expression is initially not seen in the cranial mesenchymal (cm) cells at E11 (J') but low level expression can be detected in the transgenic mesenchyme at E13 (L'). Scale bar: 100 μm in A-L'.

*Fgf9 *has been reported to be expressed in cranial mesenchymal cells as well as in the dural mesoderm during embryonic development [16]. Expression levels of the *Fgf9 *transgene and endogenous *Fgf9 *were compared by in situ hybridizations using an [^35^S]-labelled *Fgf9 *riboprobe (Fig. 6A–D'). At E11 and E13, *Fgf9 *was not expressed at detectable levels in the cranial mesenchymal cells in nontransgenic mice. In contrast, *Fgf9 *expression was clearly present in the transgenic mice (Fig. 6A–D').

As FGF9 binds to and signals through FGFR2 and FGFR3, in situ hybridizations were performed to examine their expression levels (Fig. 6E-L'). Low levels of *Fgfr2 *expression can be seen in cranial mesenchymal cells at E11 in both the nontransgenic and transgenic mice (Fig. 6E, E', F and 6F'). *Fgfr2 *expression is maintained in the induced chondrocytes in the transgenic mice (Fig. 6H, H'). In contrast, *Fgfr3 *expression was not detected at E11 either in transgenic or in nontransgenic cranial mesenchyme (Fig. 6I, I', J, J'). *Fgfr3 *was expressed at low levels in the differentiating chondrocytes of the FGF9 transgenic mice (Fig. 6L, L'). In addition, crossing the OVE1070 mice with FGFR3 null mice [14] does not alter the "dome-head" phenotype (data not shown). These results taken together suggest that the developmental switch in the transgenic mice may initially be mediated by ectopic activation of FGFR2.

### FGF9 induces proliferation of the cranial mesenchymal cells

**Figure 7:**
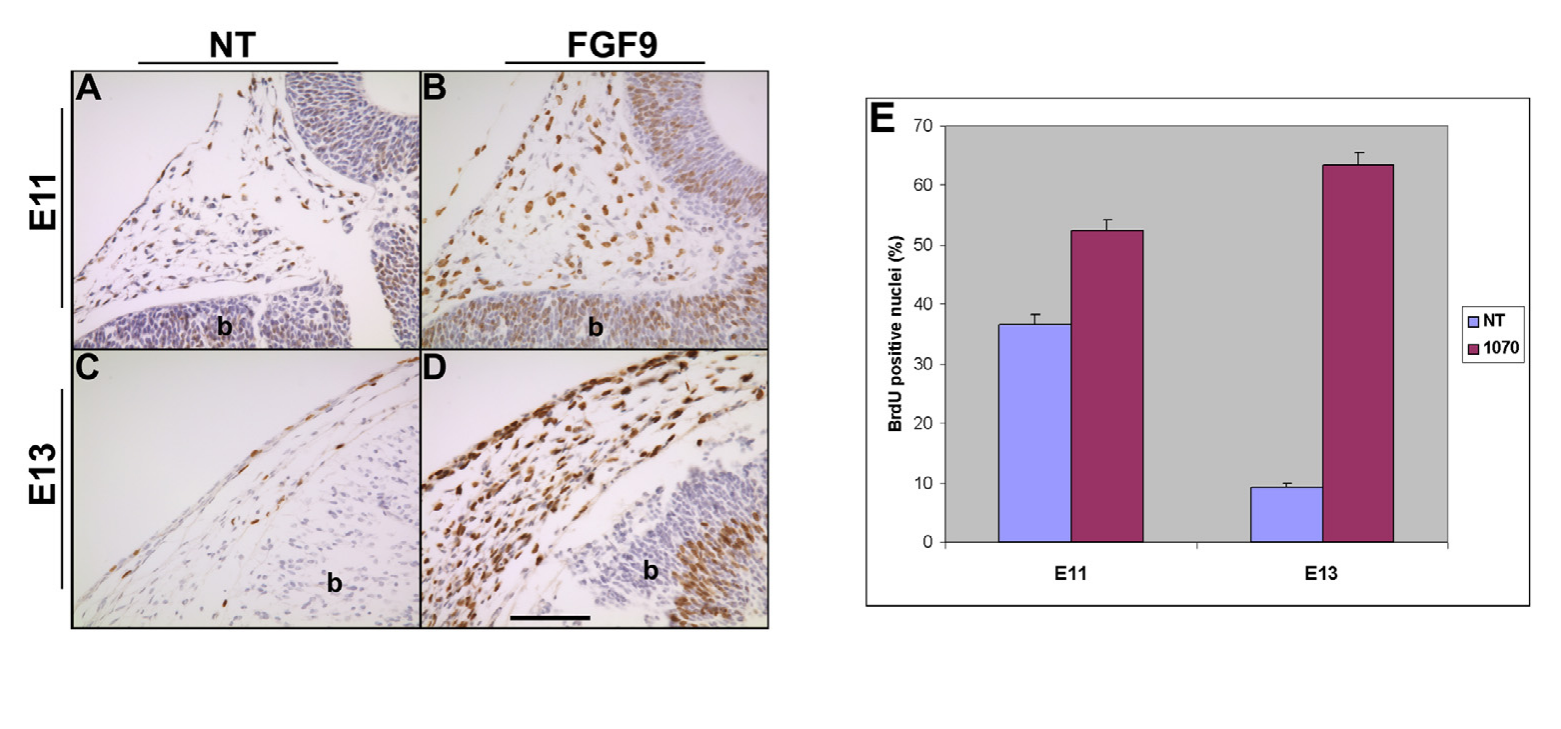
BrdU incorporation and cell proliferation. BrdU incorporation (brown stain) was detected by immunohistochemistry (A-D). A significant increase in BrdU positive (brown stained nuclei) cells was detected in the parietal region of the FGF9 transgenic mice at E11 and at E13 (E). Scale bar: 100 μm in A-D.

To test if the cranial mesenchymal cells proliferate in response to FGF9 expression, sections of E11 and E13 skulls were assayed for BrdU incorporation (Fig. 7). At E11 and at 13, the cranial mesenchymal cells in the parietal region of FGF9 transgenic mice show a significant increase in BrdU incorporation (Fig. 7A–D). These results suggest that FGF9 expression in the cranial mesenchymal cells results in increased proliferation.

### Fate mapping of cells in the parietal region in the FGF9 transgenic mice

**Figure 8:**
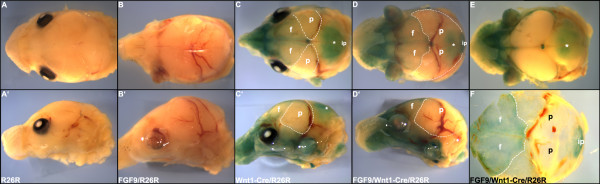
Fate mapping studies in the OVE1070 cranium. X-Gal staining of E17 embryos. Panels A-D are top views and panels A'-D' are lateral views of intact heads. In the absence of Wnt1-Cre, *lacZ *positive cells were not detected in the R26R embryo (A, A') or the FGF9/R26R embryo (B, B'). In the Wnt1-Cre/R26R embryo (C, C'), the parietal region (p) of the skull shows no evidence for *lacZ *activity, implying that neural crest cells do not contribute to this region of the skull. In the FGF9/Wnt1-Cre/R26R embryo (D, D'), there is also no blue staining in the (enlarged) parietal region. In order to distinguish the staining in the skull (E) from the staining in the underlying brain (F, asterisk), the calvarium was removed intact from embryo D and the two parts of the head are shown as top views (E, F). Since the dorsal retinal pigmented epithelium in the FGF9 transgenic mice transdifferentiates into the neural retina [30], pigmentation is seen only in the ventral half of the eye (B', arrow). Abbreviations: f, frontal; ip, interparietal.

Cell lineage tracing studies performed in mice and in chicks show that the cranial skeletogenic mesenchyme is derived from two sources, neural crest and mesoderm [35-37]. To determine if the cells in the parietal region that form ectopic cartilage in the OVE1070 mice are neural crest or mesodermal derivatives, we performed fate mapping experiments using Wnt1-Cre and R26R mice [38,39]. As has been described previously, Wnt1-Cre/R26R compound heterozygotes stably express β-galactosidase in neural crest cells and their descendants but not in the mesodermal derivatives [40,41]. OVE1070 mice were crossed with Wnt1-Cre/R26R mice and heads of E17 embryos were stained with X-Gal (Fig. 8). In the absence of Wnt1-Cre, *lacZ *positive cells were not detected in the R26R embryo (A, A') or in the FGF9/R26R embryo (B, B'). In the Wnt1-Cre/R26R embryo (C,C'), the parietal region (p) of the skull showed no evidence for *lacZ *activity, implying that neural crest derivatives do not contribute to this region of the skull. In the FGF9/Wnt1-Cre/R26R embryo (D, D'), there is also no blue staining in the (enlarged) parietal region. These results establish that the cells in the parietal region that form ectopic cartilage in the OVE1070 mice are not neural crest derived but are for the most part, derived from mesoderm. In summary, in family OVE1070, ectopic FGF9 expression switches the differentiation and proliferation properties of cranial mesodermal cells, resulting in inhibition of intramembranous ossification and conversion to a program of endochondral ossification.

## Discussion

In this study, we have analyzed a line of transgenic mice where the differentiation program of a portion of the cranial mesenchyme is converted from intramembranous to endochondral ossification. The transgenic mice ectopically express FGF9 in their cranial mesenchymal cells. Expression of FGF9 correlates with localized proliferation of the mesenchymal cells and with upregulation of *Sox9 *expression and downregulation of *CbfaI *and *Osteocalcin*. The mesenchymal cells consequently differentiate into chondrocytes that express *Col2a1*. These chondrocytes proliferate, then hypertrophy, express *Col10a1*and are later replaced by trabecular bone and bone marrow. Therefore, parietal bone formation in these mice recapitulates the sequence of events that occurs during endochondral ossification. These results demonstrate that FGF9 expression is sufficient to convert the differentiation program of (at least a subset of) the cranial mesenchymal cells from intramembranous to endochondral ossification.

### Ectopic expression of the transgene

The skeletal defects in this transgenic line (OVE1070) occur in mice that are either heterozygous or homozygous for the FGF9 transgene. It is, therefore, unlikely that the skull defects in this line of mice are due to disruption of a gene essential for membranous ossification of the parietal bones. The transgene expression pattern provides compelling evidence that the skull defects in these mice are due to ectopic expression of the transgene. In situ hybridization analyses show that the transgene is expressed in an appropriate spatial and temporal pattern to induce the developmental changes. In addition, the transgene is not expressed in regions where membranous ossification still occurs (Fig. 3G). Furthermore, transgene expression precedes *Sox9 *expression and condensation of the mesenchymal cells. Also, our results are consistent with the results of other studies. For example, recent studies of a mouse model for Apert syndrome, with a single amino acid change in FGFR2, support the notion that enhanced activation of FGFR2 can cause some cranial mesenchyme to convert to a chondrocyte differentiation program [10]. In addition, FGF9 expression in a chondrocytic cell line is sufficient to induce expression of *Sox9 *[42]. Addition of FGF-2 in vitro to cranial mesenchymal cells from quail embryos can induce cartilage differentiation at high doses [17]. Taken together, these results suggest that initiation of chondrogenesis in the cranial mesenchymal cells in this line of mice is due to ectopic expression of the FGF9 transgene.

In the OVE1070 family, expression of the FGF9 transgene is not only ectopic but also transient. Expression in the cranial mesenchyme was not detectable after E15. This implies that stimulation by FGF9 is sufficient to induce (competent) cranial mesoderm to switch to a chondrocytic differentiation program, but sustained expression of FGF9 is not required for the later stages of differentiation. Therefore, the endochondral ossification program, once initiated by FGF9, appears to become autonomous.

The reason for the ectopic *Fgf9 *expression is not clear at present. It is possible the transgene array has integrated in the neighborhood of an endogenous enhancer that directs expression to the cranial mesenchymal cells. Some of the integrated copies of the transgene still retain the αA-crystallin promoter since the transgene is still expressed in the lens [30]. The transgene is also expressed in the dorsal margins of the retinal pigmented epithelium (RPE) and this expression transforms the RPE into neuroretina [30].

### Nature of FGF9 induction

Expression of FGF9 in growth plate chondrocytes using the *Col2a1 *promoter results in reduced proliferation and terminal differentiation of chondrocytes [43]. In addition, targeted deletion of FGFR3, one of the receptors through which FGF9 signals, results in mice with overgrowth of the long bones. These results suggest that a primary role of FGF signaling in the long bones is to act as a negative regulator of chondrocyte proliferation. These results are in contrast to our results that show that ectopic FGF9 expression in embryonic cranial mesenchymal cells induces overproliferation followed by endochondral ossification. How can these disparate observations be reconciled? One possible explanation is the differential timing of FGF9 expression. The *Col2a1 *promoter is active in the differentiated chondrocytes, while our transgenic FGF9 is expressed in undifferentiated cranial mesenchymal cells. Second, there are differences in the responding tissues. Chondrocytes in the long bones originate from the lateral plate mesoderm while the parietal mesenchyme is derived from cranial mesoderm. Since the stimulated cells have different developmental origins, there is no reason to expect that the responses will be identical. For example, although lens and corneal epithelial cells are related to each other, both morphologically and developmentally, they respond differently to FGF stimulation [29,44]. The reduction in proliferation in the long bones in response to FGF stimulation may be a unique property of the growth plate chondrocytes [4]. In contrast, the FGF9-induced proliferation of cranial mesenchymal cells is consistent with the notion that FGFs, in general, act as mitogens during normal development.

### Endogenous role for FGF9?

Though FGF9 appears to be sufficient to induce chondrogenesis in the skull, the role of FGF9 during normal development of cartilage in the cranium is unclear. Our in situ hybridizations did not show any detectable expression of *Fgf9 *in the calvarial mesenchyme at E11 or E13. Expression is seen later during embryonic development in the endocranial portions of the mesenchyme and is downregulated during postnatal development [16]. This expression pattern suggests that FGF9 is not important for initial specification of the cells in the chondrocytic lineage in the cranium. Consistent with this model, targeted deletion of FGF9 does not result in any visible skeletal abnormalities in the skull [24,25]. In contrast, in the transgenic mice, the ectopic FGF9 plays an instructive role and initiates chondrogenesis. FGF9 is known to signal through FGFR2 and FGFR3 [45]. Signaling by FGF9 in the transgenic heads is likely to be mediated initially through FGFR2 rather than FGFR3 as the parietal fate switch in the FGF9 transgenic mice is not rescued by the loss of FGFR3. After Sox9 expression has been induced and the chondrocytic program has been initiated (by E13), expression of the FGF9 transgene is no longer required. In contrast to the normal chondrocytic differentiation program in long bones, ectopic chondrogenesis in the cranium proceeds at a much slower rate. Though the reasons for this are presently unclear, we speculate that this may be due to the multi-step nature of the endochondral differentiation program. Respecification and reprogramming of the parietal environment is required, and this is accomplished in a less timely fashion than at the normal sites of differentiation.

### FGFRs and intramembranous ossification

Our findings are in apparent conflict with some of the current models for the roles of FGF receptors in intramembranous ossification. Autosomal dominant mutations in FGFR2 or FGFR3 lead to premature differentiation and fusion of the skull sutures in humans and these effects are thought to be the consequence of enhanced receptor activity [9]. Based upon such a model, our transgenic mice would be predicted to exhibit an analogous phenotype, i.e. craniosynostosis. Inappropriate activation of the receptors in the cranial mesenchyme would be predicted to lead to premature differentiation. However, our studies indicate that ligand-mediated activation of FGFR2 induces proliferation of embryonic cranial mesenchymal cells and subsequent differentiation into cartilage. By extrapolation then, our results imply that mutations in the FGFR2 gene in human patients do not result in ligand-independent constitutive activation of the receptor. This prediction is supported by the finding that targeted inactivation of FGFR2 in mice causes developmental alterations in cell types that are not affected by the putative gain-of-function mutations. An alternative explanation is that transient stimulation of FGFR2 (as seen in our FGF9 transgenic mice) may lead to a qualitatively different cellular response compared to sustained ligand-independent stimulation of FGFR2 (as seen in the gain-of-function mutations in human patients). In either case, our results show that a single FGF can function as an instructive signal, inducing a specific cell type to switch from one differentiation program to an autonomous, alternative differentiation pathway.

## Conclusion

Although the mechanistic details of FGF9 stimulation of the cranial mesenchymal cells remain to be elucidated, the OVE1070 transgenic mice provide the first demonstration in vivo that an FGF can switch the differentiation program of immature cranial mesoderm. These results also demonstrate that mesoderm-derived cranial mesenchymal cells are developmentally flexible and can undergo either intramembranous or endochondral ossification in response to extracellular signals. These transgenic mice provide a model system in which to elucidate the molecular connection between stimulation of an FGFR (FGFR2) and induction of expression of a cell-fate-determining transcription factor (Sox9).

## Methods

### Generation of FGF9 transgenic mice

The construction of the FGF9 transgene and the generation of transgenic mice have been described previously [29]. The coding region of mouse *Fgf9 *(a gift from Dr. David M. Ornitz, Washington University School of Medicine, St. Louis) was inserted between the αA-crystallin promoter [46] and the SV40 small t intron/polyadenylation sequences of the CPV2 vector [47]. FGF9 transgenic mice were identified by isolating genomic DNA and screening by PCR, using primers specific to the SV40 portion of the transgene: 5'-GTGAAGGAACCTTACTTCTGTGGTG-3' (SV40A) and 5'-GTCCTTGGGGTCTTCTACCTTTCTC-3' (SV40B). The PCR cycle conditions were as follows: denaturation at 94°C for 30 seconds, annealing at 58°C for 30 seconds and extension at 72°C for 60 seconds, for 35 cycles. A final extension step of 72°C for 10 minutes was included.

### Skeletal preparations

Skeletal preparations were performed as described previously [48]. Briefly, embryos were first skinned, and eviscerated, then fixed in 95% alcohol. After fixation, the samples were stained with Alcian blue, for 1–2 days. The samples were then destained in 95% ethanol for eight hours, and cleared in 2% potassium hydroxide from eight hours to overnight depending on the size of the specimen. After clearing, the samples were stained in Alizarin red/1% potassium hydroxide overnight. The samples were cleared in 1% potassium hydroxide and 20% glycerol/1% potassium hydroxide for 2–3 days. Subsequently, the samples were allowed to harden in 1:1 95% ethanol/glycerol for one day and then transferred to absolute glycerol for storage and photography.

### Histological analyses

For routine histology, embryos were obtained from timed pregnancies using FVB/N females that were mated to heterozygous FGF9 transgenic males. Embryos were delivered by Caesarean section, fixed in 10% formalin, dehydrated, embedded in paraffin, sectioned (5 μm) and stained with hematoxylin and eosin.

### Section in situ hybridizations

To analyze the expression of different markers, in situ hybridizations were performed. Mouse cDNA clones for *Col2a1*, *Col10a1*, *Ihh *and *Sox9 *were obtained from Dr. Benoit DeCrombrugghe (University of Texas, MD Anderson Cancer Center, Houston). Mouse cDNA clones for *CbfaI *and *osteocalcin *were obtained from Dr. Gerard Karsenty (Baylor College of Medicine, Houston). cDNAs clones for *Fgf9, Fgfr2 *and *Fgfr3 *were obtained from Dr. David Ornitz (Washington University School of Medicine, St. Louis). To analyze expression of the FGF9 transgene, a [^35^S]-UTP-labeled riboprobe specific to the SV40 sequences of the transgene was made (see Fig. 1). To assay for endogenous *Sox9 *expression, a *Sox9 *antisense probe was synthesized using a *Hind*III-digested mouse *Sox9 *cDNA and T7 RNA polymerase (Promega). For *Col2a1*, the antisense probe was synthesized using *Eco*RI-digested DNA and T3 RNA polymerase. The antisense probe for *Col10aI *was synthesized using a *Hin*dIII-digested mouse *Col10a1 *cDNA and T3 RNA polymerase. The antisense probe for *Ihh *was synthesized using a *Bam*HI-digested mouse *Ihh *cDNA and T7 RNA polymerase. The antisense probe for *Fgf9 *was synthesized using a *Kpn*I-digested mouse *Fgf9 *cDNA and SP6 RNA polymerase. The antisense probe for *CbfaI *was synthesized using an *Eco*RI-digested mouse *CbfaI *cDNA and T7 RNA polymerase. The antisense probe for *osteocalcin *was synthesized using *Bam*HI-digested mouse osteocalcin cDNA and T3 RNA polymerase. The antisense probe for *Fgfr2 *was synthesized using an *Eco*RI-digested mouse *Fgfr2 *cDNA and T7 RNA polymerase. The antisense probe for *Fgfr3 *was synthesized using a *Hin*dIII-digested mouse *Fgfr3 *cDNA and T3 RNA polymerase. In situ hybridizations on tissue sections were done using hybridization and washing conditions described previously [49]. The hybridized slides were soaked in Kodak NTB-2 emulsion, dried and exposed for 3–5 days at 4°C. Following development and fixation, the slides were lightly counterstained with hematoxylin.

### Proliferation assay

DNA replication was examined by BrdU incorporation as described previously [50]. Cell proliferation was analyzed by counting the number of BrdU positive nuclei from more than 100 cells in a defined area in the parietal region in three serial sections from four (E11) or six (E13) nontransgenic and FGF9 transgenic heads. The counts for BrdU-positive cells in nontransgenic and transgenic mice were compared using the *t*-test (p < 0.01).

### Whole mount in situ hybridizations

For whole mount in situ hybridizations, tissue samples were washed thrice in PBS and fixed in 4% paraformaldehyde. Hybridizations were performed using digoxygenin-labeled sense or antisense SV40 riboprobes following standard procedures [51].

### Histochemical detection of β-galactosidase activity

Embryos were obtained from timed pregnancies of R26R homozygote females mated to OVE1070/Wnt1cre double transgenic males [36]. X-Gal staining was performed as described previously [44]. Briefly, heads of E17 embryos were collected and fixed for 2 hours at 4°C in 0.1 M phosphate buffer (pH 7.3) containing 2% paraformaldehyde, 0.2% glutaraldehyde. Following fixation, the tissue samples were rinsed thrice at room temperature in 0.1 M phosphate buffer (pH 7.3) containing 0.01% sodium deoxycholate, 0.02% NP-40, 2 mM MgCl_2_, then stained in an X-gal substrate solution (0.01% sodium deoxycholate, 0.02% NP-40, 2 mM MgCl_2_, 5 mM potassium ferricyanide, 5 mM potassium ferrocyanide, 1 mg/ml X-gal in 0.1 M phosphate buffer (pH 7.3)).

## Authors' contributions

VG designed and performed all the experiments and drafted the manuscript. PAO originated the idea to generate the CPV2-FGF9 mice, helped in the design of the experiments and the interpretation of the data and edited the manuscript. Both authors read and approved the manuscript.
